# Effects of gentle mechanical skin stimulation on subjective symptoms and joint range of motions in people with chronic neck and shoulder discomfort

**DOI:** 10.1186/s12576-023-00862-8

**Published:** 2023-03-29

**Authors:** Nobuhiro Watanabe, Marina Nara, Shihoko Suzuki, Masamitsu Sugie, Takao Yamamoto, Harumi Hotta

**Affiliations:** 1 Department of Autonomic Neuroscience, Tokyo Metropolitan Institute for Geriatrics and Gerontology, 35-2 Sakaecho, Itabashi-ku, Tokyo, 173-0015 Japan; 2 Japanese Association for Healthy Life Expectancy, Tokyo, 173-0014 Japan; 3 NHK (Japan Broadcasting Corporation), Tokyo, 150-8001 Japan; 4 Oyama Kenko-no-Machi Clinic, Tokyo, 173-0014 Japan; 5 Health Promotion Management Office, Tokyo Metropolitan Institute for Geriatrics and Gerontology, Tokyo, 173-0015 Japan

**Keywords:** Neck and shoulder discomfort, Self-care, Skin stimulation, Pain, Discomfort, Difficulty in moving, Range of motion, Acupuncture

## Abstract

This study aimed to examine the efficacy of a 2-week self-administered gentle mechanical skin stimulation on chronic neck and shoulder discomfort. In participants (*n* = 12) with chronic neck and shoulder discomfort, subjective measures of pain sensation, discomfort, and difficulty in moving using a visual analog scale (VAS, 0–10) and objective measures of 12 different joint range of motions (ROMs) for the cervical and shoulder regions, using a digital goniometer, were collected before and after self-care with contact acupuncture, called microcones. The self-care for 2 weeks significantly (*p* < 0.001) decreased all VAS scores to 2.2–2.3 from baseline values of 6.0–7.4. Of the 12 ROMs tested, 8 were significantly increased (*p* < 0.013). This open-label study suggests the use of self-care with microcones in improving subjective symptoms and joint ROMs in people suffering from chronic neck and shoulder discomfort. However, a randomized, double-blind, controlled clinical trial is needed to further investigate the efficacy and safety of microcones.

## Background

The somatosensory system processes information on stimuli to the body and plays an important role in sensation and recognition. In addition, somatosensory stimuli elicit various physiological responses, such as motor functions, autonomic functions, and hormonal secretions [1–9] as well as analgesia [10–12]. It is well-established that rigorous and consistent physiological responses are evoked by strong, noxious stimulation [8, 13–16]. In contrast, the physiological role of gentle mechanical skin stimulation, other than sensation and recognition, has been underestimated. Nevertheless, we know empirically that putting our hands on the affected area relieves pain.

To imitate continuous skin touch with a fingertip, stimulation discs, which have fine brushes called “microcones”, have been developed as inspired by non-penetrating, contact acupuncture, i.e., Japanese pediatric acupuncture [17, 18]. Skin stimulation in anesthetized rats with microcones selectively suppressed the reflex response of the cardiac sympathetic nerve by unmyelinated C-afferent excitation in the hindlimb [19]. Such a suppressive effect was similar to that of morphine [20]. This gentle skin stimulation induced low-frequency activities of cutaneous afferents with a low mechanical threshold and inhibited nociceptive transmission by activating μ-opioid systems in the spinal cord [6]. The same stimulation also suppressed the cardiovascular responses evoked by acute noxious stimulation experimentally applied to a lower limb in conscious humans [21].

Chronic neck and shoulder discomfort is one of the most common symptoms, and the prevalence in the Japanese population is reportedly 8.7% according to the National Survey of Basic Living Conditions in 2019 [22]. Clinically, somatosensory stimulation, such as acupuncture and electrical stimulation, is used to relieve neck pain [23]. One of the underlying mechanisms of such physical therapy is endogenous opioid system activation, which is in common with the microcone stimulation. Therefore, we hypothesized that the gentle mechanical skin stimulation with microcones is applicable to symptoms of neck and shoulder discomfort.

This study aimed to investigate the efficacy of a 2-week self-administered gentle mechanical skin stimulation with microcones on subjective and objective symptoms of chronic neck and shoulder discomfort. As objective symptoms, range of motions (ROMs) of the neck and shoulder regions, which are known to be decreased in people with chronic neck and shoulder discomfort [24, 25], were evaluated.

## Methods

### Participants

This study included individuals who cooperated in a survey conducted by Japan Broadcasting Corporation (Nippon Hoso Kyokai [NHK]). We received anonymized data (e.g., the intensity of symptoms of neck and shoulder discomfort, ROMs of joints, etc.), and conducted secondary data analysis. The survey implementation and anonymized data utilization in the present study are in line with the Ethical Guidelines for Medical and Biological Research Involving Human Subjects established by Japanese government organizations in 2022. This study was approved by the institutional ethical review board of Tokyo Metropolitan Geriatric Medical Center (project identification number; R22-056).

Twelve participants were enrolled, between 27 and 62 years old of both sexes, who provided informed consent including the use of the collected data for scientific research. Inclusion criteria were: persistent or repeated neck and shoulder discomfort for at least 3 months, willingness to participate, ability to come to the study site for 2 examinations before and after the self-care, and ability to use a gentle skin stimulation. Of the 70 potential participants recruited for the study, 12 met these criteria. The sample size was estimated based on the variability of subjective pain intensity in adults with chronic neck and shoulder discomfort [26] and a clinically meaningful reduction in subjective pain intensity (VAS score of ≥ 2 points) [27–29] using the G*Power software program (ver. 3.1.9.7, Heinrich-Heine-Universität Düsseldorf, Düsseldorf, Germany). To detect differences in subjective symptoms before and after 2 weeks of self-care using microcones with 90% power and a two-sided significance level of 5%, the minimum required group size was 11 participants.

### Study design, protocol, and setting

This open-label trial evaluated the efficacy and safety of the 2-week self-care with gentle mechanical skin stimulation for neck and shoulder discomfort symptoms. All participants visited the study site at baseline and after the 2-week self-care. Participants received the same assessment before and after the self-care.

**Fig. 1:**
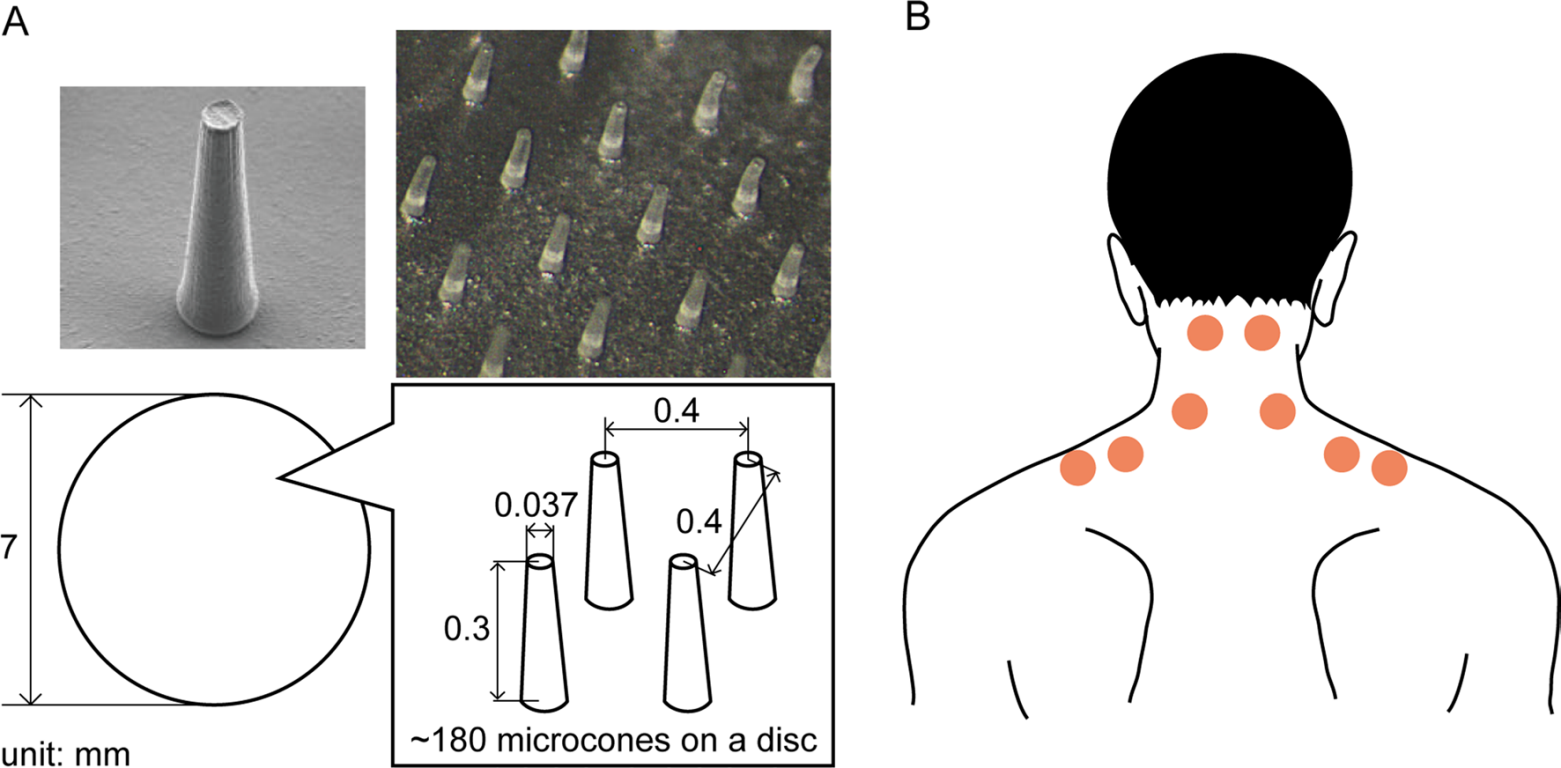
Photographs and specification of a disc with microcones (**A**) and locations of microcone application (**B**). In panel (**A**), photographs of microcones taken by scanning electron microscopy (left top) and stereoscopic microscopy (right top) are presented, and a specification of microcones is shown in the enlarged view. Approximately 180 microcones are arrayed on a circular disc of 7 mm in diameter. The microcones were regularly arrayed on a disc with a pitch of 0.4 mm. Each microcone has a flat tip with a diameter of 0.037 mm and a height of 0.3 mm. The disc was fixed on the skin with adhesive tape. In panel (**B**), filled circles indicate the approximate locations of microcone application (up to eight locations on the left and right). The locations were slightly adjusted depending on participants’ perceived symptoms and palpations performed by a physician

Skin stimulation was applied using discs with microcones made of soft elastomer, which are commercially available (Somareson L, Toyoresin Co., Shizuoka, Japan) and approved by Japanese Pharmaceuticals and Medical Device Agency as a general medical device (approval number: 22B3X10002000002). One disc has approximately 180 microcones on a surface diameter of 7 mm, with adhesive tape to be fixed on the skin (Fig. 1A). The microcones were regularly arrayed on a disc with a pitch of 0.4 mm. Each microcone has a flat tip with a diameter of 0.037 mm and a height of 0.3 mm. Immediately after measuring baseline levels, participants received individualized instruction on how and where to apply the discs. A maximum of 8 discs were put onto 8 different locations each day (anywhere on the right and left shoulders and neck), either by the participants themselves or by their family members. The locations to apply discs with microcones were determined based on participants’ subjective symptoms and palpation by an experienced physician to pinpoint stiff muscles and tender points. A handout showing the location of the microcone application was given to each participant. Approximate locations to apply microcones are presented in Fig. 1B. The participants applied the disc initially according to the handout and were also advised to adjust the locations and the number of applications as participants’ symptoms (stiffness or discomfort) changed, due to a previous finding that the gentle skin stimulation was the most effective when applied to the same or adjacent areas as the noxious input [19]. We advised removal within 12 h of application to prevent “skin rash”, although the application could be done at any time of the day. Participants were instructed to immediately discontinue the application if itching or other skin problems occurred.

### Outcome measures

This study assessed both subjective and objective symptoms associated with neck and shoulder discomfort.

Subjective symptoms were assessed using the visual analog scale (VAS), which consists of a 10-cm horizontal straight line with 0 at one end and 10 at the other end. The VAS defined 0 as no pain, no discomfort, and no difficulty in moving and 10 as the maximum imaginable pain, maximum imaginable discomfort, and maximum imaginable difficulty in moving based on pain, discomfort, and difficulty in moving, respectively. Participants rated their condition on the day of testing by marking a vertical line.

As an objective measure, joint ROM was assessed using a digital goniometer (easyangle, Ito Co., Ltd., Tokyo, Japan) for the cervical joint (flexion, extension, lateral flexion, and rotation), shoulder joint (flexion, external rotation, internal rotation, and abduction), and shoulder girdle (flexion, extension, elevation, and depression). The participants moved their joints, and ROMs were measured by an experienced physical therapist. The physician (MS) attended the measurement by the therapist (MN) and confirmed that the participants did not have active shoulder arthritis or active cervical spondylosis. The measurement was based on a standardized method [30], and care was taken not to include compensatory movements. For example, participants sat erect in the chair with their heads in neutral positions before the cervical joint movements were measured. For neck flexion and extension, the angles formed by the vertical line passing through the head’s vertex and the acromion (the reference axis) and the line passing through the vertex and external auditory canal (the motion axis) were measured. For the neck’s lateral flexion, the angles formed by the vertical line passing through the vertex and S1’s spinal process (the reference axis) and the line passing through the vertex and C7’s spinal process (the motion axis) were measured. For the neck’s rotation, the angles formed by the perpendicular line crossing the line connecting both acromia (the reference axis) and the line passing through the nose bridge and occipital protuberance (the motion axis) were measured. The shoulder’s flexion and abduction movements were measured as the angles formed by the vertical lines through the acromion (the reference axis) and the humerus (the motion axis). The shoulder’s external and internal rotations were measured as the angles formed by the line that passes perpendicularly onto the coronal plane (the reference axis) and the ulna (the motion axis), with the elbow joint flexed at 90°. The flexion and extension movements of the shoulder girdle were measured as the angles formed by the line connecting both acromia (the reference axis) and the line passing through the head’s vertex and the acromion (the motion axis). The elevation and depression movements were measured as the angles formed by the line connecting both acromia (the reference axis) and the line passing through the rostral edge of the sternum and the acromion (the motion axis). Each movement was measured once because repeated joint ROM measurements on the same day will gradually improve the results. The left and right values were averaged for items that included both right and left movements.

Safety was assessed by recording adverse events.

### Statistical analysis

Prism 9 software (GraphPad Inc., La Jolla, CA, USA) was used for statistical analysis. Data obtained before and after the self-care periods were compared using a paired *t* test or Wilcoxon matched-pair signed rank test. The correlation between VAS and ROM changes and their baseline values or participant age and between VAS score and ROM changes following the self-care period was tested by Pearson correlation coefficients or Spearman correlation. VAS and ROM changes were compared between males and females using unpaired *t* test. The statistical test was based on the normality of the data and tested by the Kolmogorov–Smirnov test. The statistical significance level was set at 5%. Data are expressed as the mean ± standard deviation (SD) unless otherwise stated.

## Results

### Participants

**Fig. 2:**
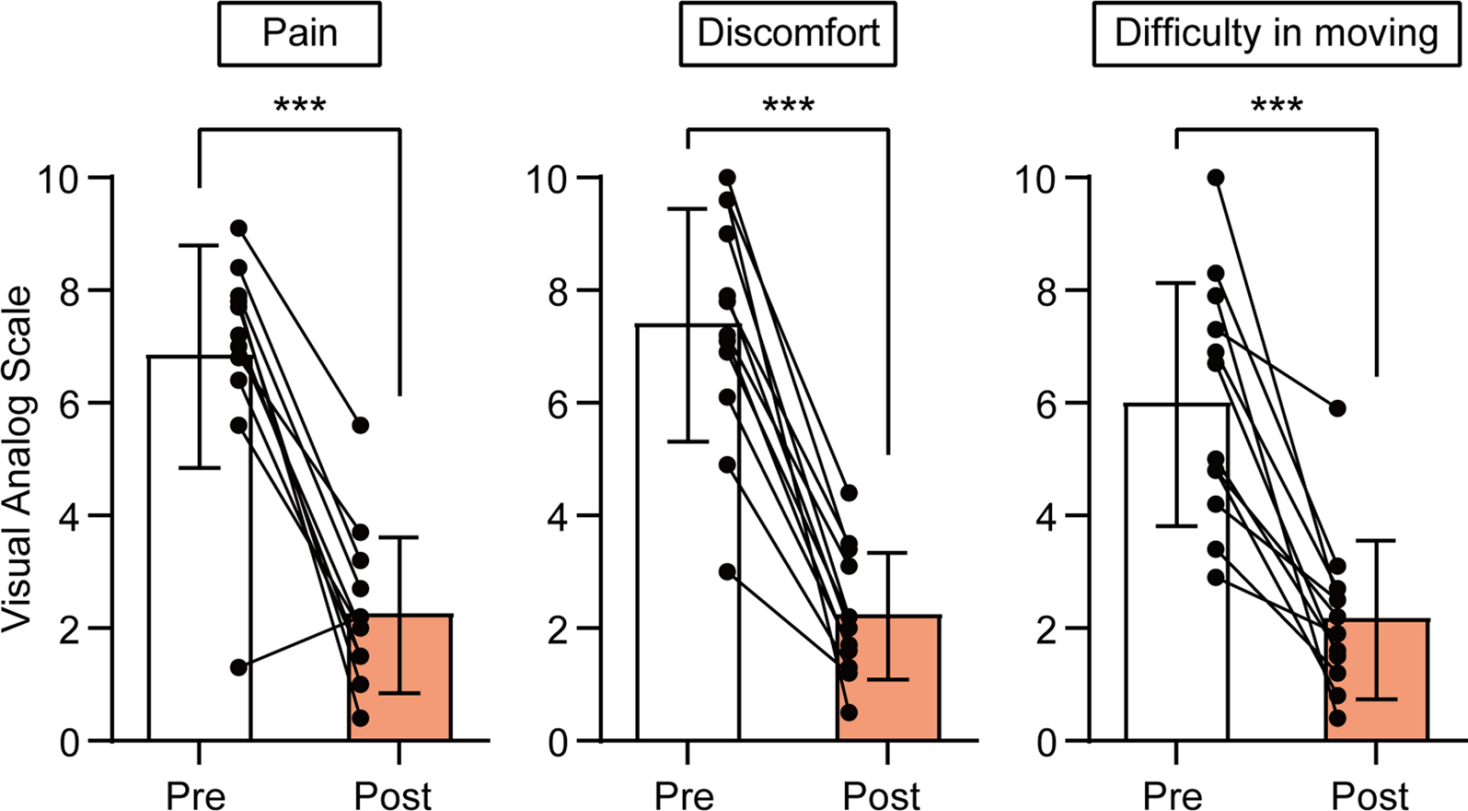
The effect of the 2-week self-care using microcones on subjective symptoms. Pain, discomfort, and difficulty in moving intensities were evaluated using a visual analog scale. 0 = no subjective symptom (pain, discomfort and difficulty moving) and 10 = the most severe symptom imaginable. Data obtained before (Pre) and after (Post) the self-care periods were compared using the paired *t* test. Asterisks indicate a significant difference (***p < 0.001). Individual closed circles and lines indicate data obtained from each participant. The bar chart and vertical bar indicate the mean and standard deviation. *N* = 12

**Fig. 3:**
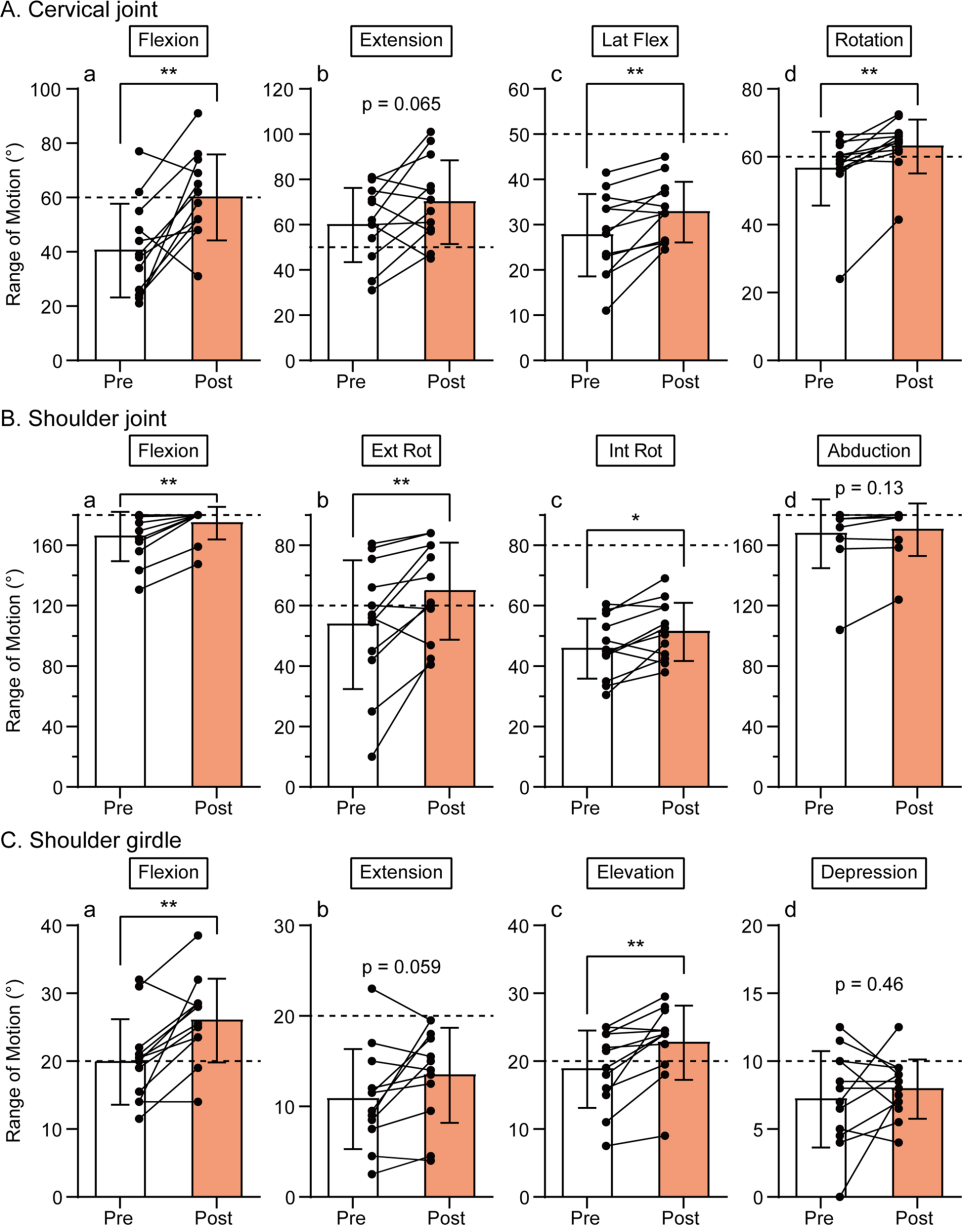
The effect of the 2-week self-care using microcones on the range of motion in the cervical joint (**A**), shoulder joint (**B**), and shoulder girdle (**C**). Data obtained before (Pre) and after (Post) the self-care periods were compared using the paired *t* test or Wilcoxon matched-pairs signed rank test. Asterisks indicate a significant difference (*p < 0.05, **p < 0.01). Individual closed circles and lines indicate data obtained from each participant. The bar chart and vertical bar indicate the mean and standard deviation. A horizontal dashed line indicates a reference value of the range of motion (Japanese Society of Rehabilitation Medicine, 2021). *N* = 12 (except shoulder joint abduction, *N* = 11). Ext Rot = external rotation, Int Rot = internal rotation, Lat Flex = lateral flexion

All 12 participants completed the study. The baseline demographic and clinical characteristics of these participants are summarized in Table 1. The mean age of the participants was 47 years old (range 27–62 years old), with 7 females and 5 males. Baseline values of subjective pain, discomfort, and difficulty in moving intensities were 6.9 ± 2.0, 7.4 ± 2.1, and 6.0 ± 2.2, respectively (see also Fig. 2). The participants had restricted ROMs in at least one direction in each joint, i.e., flexion (reference value: 60° vs. 40.9° ± 17.3°, Fig. 3Aa) and lateral flexion (50° vs. 28.0° ± 9.1°, Fig. 3Ac) of the cervical joint, shoulder joint internal rotation (80° vs. 46.3° ± 9.9°, Fig. 3Bc), and shoulder girdle extension (20° vs. 11.0° ± 5.5°, Fig. 3Cb), compared to the reference value [30].

**Table 1:** Baseline demographic and clinical characteristics of participants

Demographic data			
Number	12		
Sex [%]	Male; 5 [41.7%]	Female; 7 [58.3%]	
Age (mean [range])	47 [27–62]		

Clinical data	Mean (SD)	Minimum	Maximum
*VAS*			
Pain intensity (0–10)	6.9 (2.0)	1.3	9.1
Discomfort (0–10)	7.4 (2.1)	3	10
Difficulty in moving (0–10)	6.0 (2.2)	2.9	10
*ROMs of cervical joint*			
Flexion (°)	40.9 (17.3)	21.0	77.0
Extension (°)	60.4 (16.5)	31.0	81.0
Lateral flexion (°)	28.0 (9.1)	11.0	41.5
Rotation (°)	56.9 (10.9)	24.0	66.5
*ROMs of shoulder joint*			
Flexion (°)	166.7 (16.3)	130.5	180.0
External rotation (°)	54.2 (21.3)	10.0	80.5
Internal rotation (°)	46.3 (9.9)	30.5	60.5
Abduction (°)^†^	168.5 (22.7)	104.0	180.0
*ROMs of shoulder girdle*			
Flexion (°)	20.1(6.3)	11.5	32.0
Extension (°)	11.0 (5.5)	2.5	23.0
Elevation (°)	19.0 (5.7)	7.5	25.0
Depression (°)	7.3 (3.6)	0	12.5

ROM = range of motion, VAS = visual analog scale, SD = standard deviation

^†^Abduction datum of a participant is missing and the result of 11 participants is reported

### Efficacy

#### VAS

**Fig. 4:**
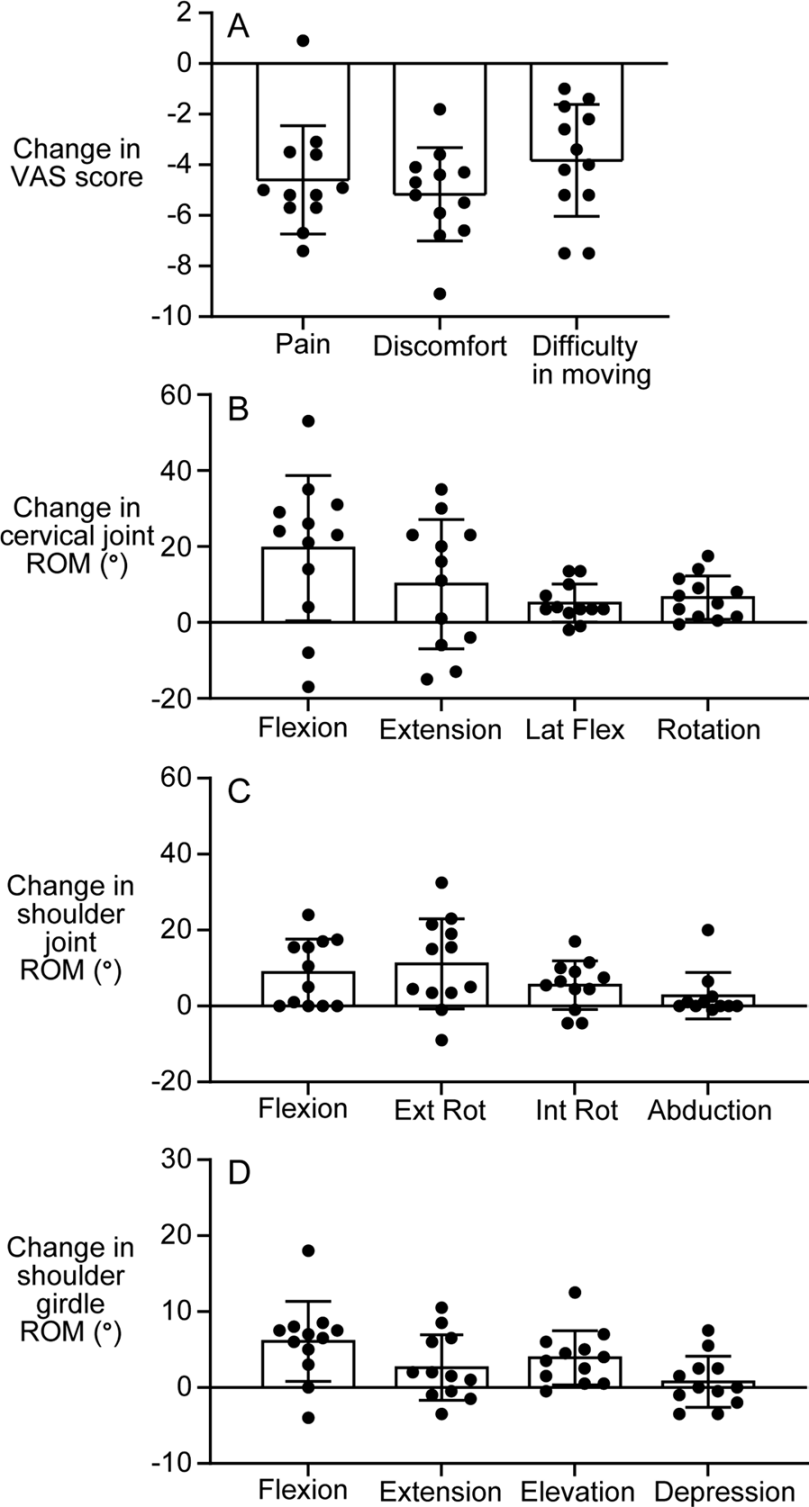
The magnitude of changes in subjective symptoms (**A**) and range of motion (**B**–**D**). Data obtained before self-care periods were subtracted from those after self-care. The individual closed circle indicates data obtained from each participant. The bar chart and vertical bar indicate the mean and standard deviation. *N* = 12. Ext Rot = external rotation, Int Rot = internal rotation, Lat Flex = lateral flexion, ROM = range of motion, VAS = visual analog scale

The 2-week self-care with microcones significantly improved subjective symptoms of neck and shoulder discomfort. The values of pain, discomfort, and difficulty in moving intensities measured by VAS after the self-care period became 2.3 ± 1.4, 2.3 ± 1.1, and 2.2 ± 1.4, respectively, and were significantly lower than the baseline values described above (*p* < 0.001, Fig. 2). This improvement was consistent across participants, except for one participant for pain intensity. The magnitude of VAS reduction for pain, discomfort, and difficulty in moving was 4.6 ± 2.1, 5.2 ± 1.8, and 3.8 ± 2.2, respectively (Fig. 4A). The magnitudes of VAS reduction were not significantly different between sexes and were not correlated with participant age. However, the VAS changes were negatively correlated with the corresponding baseline VAS values (Table 2).

**Table 2:** The correlation coefficients (r) between baseline values and changes in subjective and objective measures

Subjective measures			
Pain	Discomfort	Difficulty in moving	
− **0.78**	− **0.84**	− **0.79**	

Objective measures			
*ROMs of cervical joint*			
Flexion	Extension	Lateral flexion^$^	Rotation
− **0.62**	− 0.39	− 0.58	− **0.71**
*ROMs of shoulder joint*			
Flexion	External rotation	Internal rotation	Abduction^$,†^
− **0.80**	− **0.67**	− 0.37	− 0.57
*ROMs of shoulder girdle*			
Flexion	Extension	Elevation	Depression
− 0.44	− 0.45	− 0.38	− **0.80**

*r* = Pearson *r* (^$^Spearman *r*). −1 ≤ *r* < − 0.7: strong negative correlation, − 0.7 ≤ *r* < − 0.4: moderate negative correlation, − 0.4 ≤ *r* ≤ − 0.2: weak negative correlation, − 0.2 < *r* < 0.2: no correlation. Values in bold font; *p* < 0.05

^†^Abduction datum of a participant is missing and the result of 11 participants is reported

#### ROM

The objectively measured ROMs of the cervical and shoulder joints and shoulder girdle were generally greater after 2 weeks of self-care. Regarding the cervical joint movements, flexion, lateral flexion, and rotation, except for extension, were increased (*p* = 0.0046, 0.0045, and 0.0015, respectively, Fig. 3A). Regarding the shoulder joint movements, flexion (*p* = 0.0078), external rotation (*p* = 0.008), and internal rotation (*p* = 0.013), except for abduction, were increased (Fig. 3B). Regarding the shoulder girdle movements, flexion (*p* = 0.0021) and elevation (*p* = 0.002), but not extension or depression, were increased (Fig. 3C). Especially, some restricted motions improved to the reference values; for example, cervical joint flexion (60.5° ± 15.9°, Fig. 3Aa) and rotation (63.4 ± 8.0°, Fig. 3Ad), shoulder joint external rotation (65.3° ± 16.0°, Fig. 3Bb), and shoulder girdle elevation (22.9° ± 5.5°, Fig. 3Cc). The largest change was found in cervical joint flexion (19.6° ± 19.1°, Fig. 4B), shoulder joint external rotation (11.1° ± 11.9°, Fig. 4C), and shoulder girdle flexion (6.1° ± 5.3°, Fig. 4D). No sex difference was found in any ROM changes. However, the ROM changes in two motions, i.e., shoulder flexion (*r* = 0.61, *p* = 0.036) and shoulder internal rotation (*r* = 0.59, *p* = 0.041), were positively correlated with participant age. A significant negative correlation with the baseline ROM was found in ROM changes in 5 of the 12 motions (Table 2).

#### VAS vs. ROM

The correlation between the magnitude of changes in ROM (Δ°) and those in subjective measures (Δ points) was examined. Negative correlations were found between neck rotation and discomfort (*r* =  − 0.65, *p* = 0.022) and between shoulder flexion and difficulty in moving (*r* =  − 0.64, *p* = 0.024), but without statistically significant correlations from other measures (Table 3).

**Table 3:** The correlation coefficients (*r*) between changes in subjective measure and range of motion (ROM)

	Pain	Discomfort	Difficulty in moving
*ROMs of cervical joint*			
Flexion	0.035	− 0.16	− 0.15
Extension	0.49	− 0.46	0.052
Lateral flexion^$^	0.014	− 0.22	0.016
Rotation	− 0.29	**− 0.65**	− 0.32
*ROMs of shoulder joint*			
Flexion	− 0.32	− 0.32	**− 0.64**
External rotation	− 0.19	− 0.40	− 0.22
Internal rotation	0.46	− 0.11	− 0.063
Abduction^$, †^	0.42	0.16	0.24
*ROMs of shoulder girdle*			
Flexion	− 0.3	− 0.56	− 0.45
Extension	0.31	− 0.18	0.0085
Elevation	− 0.09	− 0.45	− 0.28
Depression	0.22	0.046	0.13

*r* = Pearson *r* (^$^Spearman *r*). − 0.7 ≤ *r* < − 0.4: moderate negative correlation, − 0.4 ≤ *r* ≤ − 0.2: weak negative correlation, − 0.2 < *r* < 0.2: no correlation, 0.2 ≤ *r* < 0.4: moderate positive correlation, 0.4 ≤ *r* < 0.7: moderate positive correlation. Values in bold font; *p* < 0.05

^†^Abduction datum of a participant is missing and the result of 11 participants is reported

### Safety

No adverse events were reported in 12 participants, and none discontinued the use of microcones due to adverse events.

## Discussion

### Subjective symptoms

The subjective pain intensity (VAS score) before self-care in the study participants was ≥ 4 points, except for one participant, and their symptoms were relatively severe [31]. The VAS score decreased by an average of 4.6 points due to the 2-week self-care. A decreased VAS score of ≥ 2 points is a clinically significant reduction [27–29]. Effective non-invasive treatments for non-specific neck pain include transcutaneous electrical nerve stimulation (TENS), acupuncture, and mobilization [23]. Studies that applied TENS and acupuncture for 5 days to 12 weeks reported a significant reduction in subjective pain intensity by 2.4–4.95 (calculated as an assessment of 0–10) [32–35]. The present study revealed a similar magnitude of decreased VAS, supposing that self-care using microcones is as effective as other stimulation therapies.

Previously mentioned studies used pain intensity as the main subjective measurement. The present study also assessed discomfort and difficulty in moving as subjective measures and revealed that the use of microcones attenuated these measures. Pain, discomfort, and difficulty in moving are perceived by patients with musculoskeletal conditions. Each perception includes different meanings although there are some overlaps among these perceptions [36]. Therefore, the present results indicate that multidimensional perceptions were mitigated by gentle stimulation.

Previously, we tested the effect of the microcones in healthy volunteers, wherein the application of microcones reduced cardiovascular responses to noxious heat stimulation, whereas subjective pain intensity measured using VAS was not attenuated [21]. This study has a few differences from our present study which lead to contradictory results of subjective pain intensity. First, the present study examined the effect of microcones on chronic and deep-tissue pain while acute and superficial pain in the previous study. Additionally, the frequency and duration of the stimulation were different between the present and previous studies; every day for 2 weeks vs. single use and a maximum of 12 h/day vs. 10 min, respectively.

### ROMs

The cervical joint ROMs were used as an objective measure to assess the effect of stimulation therapies, such as acupuncture, TENS, and manual therapies, on neck pain. In those studies, the cervical ROMs increased [32, 34, 37] or did not change [38]. Thus, the effect on the ROMs was not always consistent with subjective measures using VAS. The present study revealed no correlation between the magnitude of changes in VAS and ROM in each participant although VAS decreased and ROM increased in most joint motions. Therefore, such dissociation indicates that the ROM improvement was independent of the amelioration of subjective symptoms. The significance of obtaining objective measures, such as ROMs, was re-recognized, in addition to recording VAS scores, to better understand the effectiveness of interventions employed.

The trapezius muscles are reportedly stiffer in individuals with neck and shoulder discomfort than in those without such symptoms [26]. ROM is affected by muscle stiffness [39, 40], although the joint ROM is an indicator of joint function assessment. An increased neck flexion motion is the most significant among improved ROMs in the present study. The motion stretches the trapezius muscles. Hence, the present study found increased ROMs, which indicate attenuations of muscle stiffness, such as the trapezius muscles. Skeletal muscles in the neck and shoulders, including the trapezius muscles, attach to multiple joints and possibly lead to ROM increases in the shoulder joint and girdles, in addition to the cervical ROM. ROMs of the shoulder joint and girdle were improved in the present study. Therefore, the ROMs of the shoulder joint and girdle are important measures to assess neck–shoulder conditions and treatment effectiveness.

The pathophysiology of developing stiffness of the neck and shoulder muscles remains unclear. One possible explanation is attributed to the vicious circle model that nociceptive inputs induce muscle tension and afferent activity elicited by muscle contraction that enhances muscle tension [41, 42]. Additionally, muscle contraction is reflexively regulated by sympathetic nerves, leading to increased skeletal muscle contractility [2, 43]. The effect of skin stimulation with microcones in animals was abolished by transection of the skin nerves where microcone stimulation was applied [19]. Therefore, ROM increase by the gentle skin stimulation may suggest that the microcones effectively inhibit neural reflex that increase muscle tension (i.e., stiffness), including sympathetic reflex inhibitions [6, 19, 21, 44]. In contrast, there is still the possibility that ROM improvement is related to other effects including a direct effect on the muscles or a change in blood flow, because muscle stiffness and pain are also induced by factors other than neural reflex and sympathetic nerve activity.

### Physiological roles of gentle mechanical skin stimulation apart from sensation and recognition

Skin afferent inputs are considered important for sensation and recognition, however, there are also skin afferents that do not contribute to such sensory functions. Previous studies have shown that some kind of gentle mechanical skin stimulation modulates motor and autonomic functions in animals, in whom consciousness was eliminated by anesthesia and the influence of sensation and emotions were excluded [6, 19, 44–48]. We have shown that the presence or absence of microcones, that is perceptually indistinguishable, subliminally affected glucose metabolism in the anterior cingulate cortex [49], suggesting that gentle skin stimulation using microcones influences physiological functions, independently of sensation and recognition, even in conscious humans.

Electrophysiologically recording a single unit activity of skin afferents in anesthetized rats showed no difference in Aβ-afferent responses to skin stimulation with or without microcones, however, higher Aδ- and C-afferent activity with microcone stimulation [6], suggesting that low-threshold Aδ- and C-afferents, which do not contribute to sensation or recognition, are involved in various physiological functions. The analgesic effect of gentle mechanical skin stimulation was reported by Liljencrantz et al. [50]. The study showed that slow stroking stimulation decreased pain intensity induced by heat stimulation, whereas fast stroking and vibration stimulation had no effect on pain. Slow stroking stimulation was particularly effective in the excitation of low-threshold mechanoreceptive C-afferents [51, 52]. Although we did not compare the effect of skin stimulation with and without microcones in the present study, the results that gentle skin stimulation reduced both subjective and objective symptoms of chronic deep-tissue pain indicate an important physiological role of gentle mechanical skin stimulation other than sensation and recognition.

### Limitations

The present results indicate that gentle mechanical skin stimulation with microcones is a self-care method, which is effective for improving subjective and objective measures and is safe to be used. In contrast, one of the limitations of the present study is a small, non-blinded study without controls. Psychological factors such as placebo effect, and also non-specific acupressure effect cannot be excluded from the effect of microcones in this study. The flat surface disc without microcones [21, 49] may be useful as a placebo control. Therefore, comparison with a placebo control would be required in future studies. Another limitation is a potential bias of participant’s characteristics, such as socioeconomics (education history and occupation) and regional environment (access to the study site), which might influence the effectiveness of the self-care treatment. Additionally, the data collected during a follow-up period may be valuable for knowing the length of the effect of microcones following the cessation of a stimulation period. Regardless of these shortcomings of the present small study with a simple design, the present results at least suggest a potential treatment option with a non-invasive and useful method, and motivate to proceed the self-care method to a more rigorously designed study with a larger sample size. Therefore, large, multicenter, double-blind, controlled clinical trials are essential to establish the safety and efficacy of microcones for chronic neck and shoulder discomfort treatment in the future.

## Conclusion

The present results suggest microcones as a safe and effective treatment for neck and shoulder discomfort. However, this was a small, open-label study and did not include a control group. Therefore, large, multicenter, double-blind, controlled clinical trials are needed to further evaluate the safety and efficacy of microcones in the future.

## Data Availability

The original contributions presented in the study are included in the article, and further inquiries can be directed to the corresponding author.

## Abbreviations

NHK: Nippon Hoso Kyokai
ROM: Range of motion
SD: Standard deviation
TENS: Transcutaneous electrical nerve stimulation
VAS: Visual analog scale
